# Brain Stimulation for Psychiatric Disorders: Emerging Evidence and New Perspectives—2nd Edition

**DOI:** 10.3390/brainsci16070726

**Published:** 2026-07-09

**Authors:** Jacopo Lisoni, Stefano Barlati

**Affiliations:** 1 Department of Mental Health and Addiction Services, ASST Spedali Civili of Brescia, 25123 Brescia, Italy; stefano.barlati@unibs.it; 2 Department of Clinical and Experimental Sciences, University of Brescia, 25123 Brescia, Italy

The application of Brain Stimulation to psychiatric disorders has undergone a substantial transformation over the past decades. What initially emerged as a translational extension of neurophysiology has progressively developed into a clinically oriented and methodologically sophisticated field, encompassing a wide range of non-invasive and invasive interventions. Following the great success of the first edition of the Special Issue “Brain Stimulation for Psychiatric Disorders: Emerging Evidence and New Perspectives”, this second edition was conceived to reflect such evolution, bringing together contributions that span different neuromodulation modalities. Overall, the works included in this Special Issue offer a broad perspective on Brain Stimulation in psychiatry, covering non-invasive techniques such as transcranial Direct Current Stimulation (tDCS), repetitive Transcranial Magnetic Stimulation (rTMS), and Theta Burst Stimulation (TBS), as well as invasive approaches such as Deep Brain Stimulation (DBS). Here, international researchers illustrate the breadth of current research across a spectrum of different psychiatric conditions, including major depressive disorder (MDD), obsessive–compulsive disorder (OCD), schizophrenia, attention deficit hyperactivity disorder (ADHD), gambling disorder (GD) and borderline personality disorder (BPD).

Three main themes emerged from this Special Issue, providing the framework through which the included contributions are discussed in this Editorial.

The first theme considers the growing interest in the application of NIBS for BPD treatment, as demonstrated by the increasing number of systematic reviews and clinical trials recently published [1,2,3,4]. In fact, accumulating evidence suggests that BPD is associated with identifiable neurobiological alterations that can explain the clinical features of the disorder. These findings provide a compelling rationale for NIBS application in BPD, pointing toward a more circuit-based framework in which stimulation targets and outcome measures are aligned with the neurobiological substrates underlying the core psychopathological dimensions of BPD. Notably, the largest tDCS study conducted in BPD showed that anodal stimulation of the right dorsolateral prefrontal cortex (DLPFC) was associated with significant improvements in impulsivity, attentional functioning, and affective symptoms [2]. In this Special Issue, Cailhol and colleagues present a narrative synthesis of the current evidence on tDCS in BPD, integrating findings from five randomized controlled trials (RCTs): the collected studies demonstrated an effective profile of tDCS in improving emotional regulation, inhibitory control and executive functioning, rejection sensitivity, and comorbid depressive symptoms. As for the latter, these findings are in line with recent meta-analytic evidence suggesting a transdiagnostic effect of tDCS in reducing anxiety and depressive symptoms across different mental conditions [5]. Particularly, in the case of BPD, such findings are of great clinical interest since affective symptoms represent one of the most frequent comorbidities in BPD for which patients require a treatment plan. Cailhol and coworkers also addressed key practical considerations with remarkable attention to detail, including the feasibility of combining tDCS with psychotherapeutic interventions, as well as safety-related aspects. We also include a randomized, double-blind, sham-controlled study protocol by De Panfilis and colleagues investigating the feasibility and durability effects of 10-session anodal tDCS over the right ventrolateral prefrontal cortex in BPD individuals. Notably, this tDCS protocol is grounded in a circuit-based framework, aiming to align stimulation targets with the neurobiological substrates of BPD psychopathology. As preliminary evidence has suggested that 1-day stimulation in this region attenuates rejection-related emotional (RRE) responses [5], this study will evaluate the impact of tDCS on core dimensions of BPD, such as RRE responses during real-life social interactions, thereby providing a broad assessment of affective instability and interpersonal sensitivity domains. Notably, the study also incorporates ecological momentary assessment, offering the opportunity to capture emotional and interpersonal processes in everyday life rather than relying exclusively on retrospective clinical ratings. This integration of mechanistic rationale and real-world outcome assessment represents a valuable step toward tailoring NIBS interventions to the complex and dynamic clinical features of BPD.

Beyond BPD, impulsivity is a hallmark of other psychiatric conditions, including ADHD and GD, in which it is typically described at the neuropsychological level as a lack of inhibitory control and impaired executive functioning. In this Special Issue, two reports observed that NIBS can effectively enhance these cognitive functions among clinical populations. Gavazzi and colleagues assessed the effects of a single-session continuous TBS (cTBS) over the bilateral pre-supplementary motor area to improve inhibitory control in 18 patients with GD, 17 healthy volunteers, and two GD patients treated with sham-cTBS. Cognitive assessment included the stop-signal task (SST). Significant improvements in SST indices—particularly stop-signal reaction times (SSRTs)—were observed in GD patients, reflecting enhanced proactive inhibitory control. Rodríguez-Prieto and colleagues reported a single-case study testing a multimodal intervention combining 10-session prefrontal tDCS, digital cognitive training, and Cognitive Behavioral Therapy in an adult woman with predominantly inattentive ADHD. At study completion, improvements in executive functions (namely cognitive flexibility, visual working memory, and planning) were found. Moreover, meaningful reductions in depressive symptoms and improvements in quality of life were observed, whereas anxiety symptoms, sustained attention, auditory working memory, and processing speed showed limited or no change.

The second major theme of this Special Issue focuses on advancements in invasive interventions, collecting several contributions on DBS across treatment-resistant psychiatric disorders. More specifically, these studies move from descriptive efficacy to more nuanced mechanistic and clinical interpretations. For example, the comprehensive narrative review by Khosravi highlighted the broad transdiagnostic potential and safety profile of DBS, reporting clinically meaningful improvements in MDD and OCD and emerging evidence in bipolar disorder, schizophrenia, addictions, and anorexia nervosa. Moreover, this synthesis underscores key limitations of DBS research, including the lack of robust and long-term RCTs as well as the heterogeneity in stimulation targets and parameters. Similarly, Torres Díaz et al. provided a comprehensive overview of DBS in treatment-resistant psychiatric disorders, integrating clinical efficacy with emerging technological, neurobiological, and ethical perspectives. More specifically, the authors emphasize the transition toward circuit-based and connectomic approaches, highlighting the role of advanced neuroimaging in refining target selection and optimizing network engagement. The review also discusses the growing relevance of adaptive and closed-loop DBS systems, as well as the integration of artificial intelligence to personalize surgical planning and stimulation parameters. The authors also speculated on the identification of novel stimulation targets to support a shift toward more precise, symptom-specific interventions. Building on this framework, the systematic review by Pomes and colleagues provides a focused synthesis of the psychiatric and cognitive sequelae associated with subthalamic nucleus DBS (STN-DBS) in patients with Parkinson’s disease and OCD. Collecting evidence from 16 studies, the authors highlighted both short- and long-term effects, including transient activation states (e.g., hypomania, euphoria, and increased impulsivity) as well as longer-term risks, particularly increased apathy and shifts in depressive symptoms, alongside potential impairments in verbal fluency. Crucially, these outcomes appeared highly dependent on stimulation parameters as well as individual patient vulnerability, supporting the view that psychiatric changes are not incidental but linked to the modulation of brain networks in which the STN acts as an integrative node. From a clinical perspective, these findings underscore the need for systematic psychiatric assessment before and after DBS implantation, as well as close longitudinal monitoring.

A third topic of this Special Issue concerns the personalization of NIBS interventions across mental disorders, moving from the identification of patient-related moderators of clinical response to the development of neurophysiological and cellular models to capture individual variability in treatment outcomes. Considering the evaluation of patient-related variables, Lisoni and colleagues demonstrated that cigarette smoking may moderate the effects of prefrontal tDCS outcomes in schizophrenia, negatively influencing cognitive symptoms but not negative symptoms. Indeed, compared with smokers, tDCS was associated with significant improvements in working memory and verbal fluency domains only in non-smoking patients. Conversely, negative symptoms improved in both the smoking and non-smoking groups. Consistent with a recent meta-analysis [6], the authors hypothesized that chronic exposure to cigarette smoke-related neurotoxins may foster a neurotoxic milieu capable of blunting the neuroprotective and plasticity-enhancing effects of tDCS. By highlighting the role of patient-related factors in shaping treatment response, these findings support a more personalized use of tDCS in schizophrenia, whereby individual clinical characteristics may guide therapeutic decision-making and help avoid the indiscriminate application of NIBS across patients. Hodzic and colleagues addressed a crucial methodological issue for precision neuromodulation: how to reliably probe cortical plasticity in humans. In a randomized, double-blind crossover study in healthy controls, they compared four paired associative stimulation (PAS) paradigms, testing PAS-10 and PAS-25 protocols with either 180 or 225 pairings. The aim was to identify stimulation parameters capable of producing reliable LTD- and LTP-like corticospinal plasticity. Importantly, PAS-25 with 225 pairings emerged as the most robust and time-efficient protocol for inducing LTP-like increases in corticospinal excitability, whereas PAS-10 unexpectedly produced facilitatory rather than inhibitory effects. Beyond its technical implications, this study is relevant for psychiatrists involved in Brain Stimulation research as altered cortical plasticity is increasingly recognized as a transdiagnostic mechanism in disorders such as MDD and schizophrenia. Standardized and reliable PAS protocols may therefore help characterize individual differences in neuroplastic capacity, identify potential biomarkers of treatment response, and refine patient selection for NIBS interventions. Given the documented efficacy of rTMS paradigms in OCD as discussed in the most recent guidelines in the field [7], a pioneering multicenter study by Benatti and colleagues further investigated treatment response in treatment-resistant OCD by integrating clinical stratification, neuromodulation, pharmacological response profiles, and patient-derived cellular models. In detail, patients were stratified according to their response to pharmacological treatment and to either dorsal medial prefrontal cortex deep rTMS or left orbitofrontal cortex cTBS. Moreover, peripheral blood mononuclear cells were collected and reprogrammed into human-induced pluripotent stem cells and differentiated into neuronal cultures enriched in striatal medium spiny neurons in order to establish an in vitro platform to explore morphological, biochemical, and transcriptomic features associated with different treatment-response profiles. Importantly, the study proposes that cellular models may be used not only to characterize disease mechanisms but also to test biological sensitivity to therapeutic interventions. Although the reported clinical data remain preliminary, this work moves beyond symptom-based classification and supports a translational strategy in which treatment response can be investigated at the clinical and cellular levels. Additionally, to personalize NIBS intervention, a relevant topic that researchers must consider is the potential effect of psychiatric comorbidity on treatment outcomes. Within this context, Trandafir and colleagues examined the role of intermittent TBS (iTBS) in MDD with comorbid anxiety symptoms, evaluating its efficacy and tolerability profiles and identifying clinical factors that may influence treatment response. This systematic synthesis included six studies, suggesting that iTBS may produce significant improvements in both depressive and comorbid anxiety symptoms. However, the strength of the evidence remained limited by the small number of available studies, substantial heterogeneity in study designs and stimulation protocols, and the predominance of open-label trials. Nevertheless, the authors observed that treatment outcomes were largely influenced by age, baseline severity, and medication status. Particularly, antipsychotics, anticonvulsants, and benzodiazepines were associated with attenuated clinical benefit, while bupropion was associated with increased response.

Finally, this Special Issue provides some evidence from neurofeedback studies. Although neurofeedback is not a NIBS technique in the strict sense, its inclusion is justified within the broader framework of non-invasive, brain-based interventions for psychiatric disorders. Rather than directly stimulating neural tissue, EEG–fMRI-based neurofeedback provides real-time information on neural activity to promote learned self-regulation of clinically relevant circuits. Accordingly, the studies by Amital and colleagues on reward-system neurofeedback in MDD and anhedonia and by Tendler and coworkers on amygdala-derived neurofeedback for PTSD-related sleep disturbances should be regarded as complementary exploratory contributions, extending the discussion from externally applied stimulation to feedback-guided modulation of dysfunctional brain networks.

In conclusion, this second Special Issue once again highlights the broad scope of invasive and non-invasive Brain Stimulation approaches in psychiatry, emphasizing their potential to improve clinical symptoms and foster personalized therapeutic strategies across mental disorders. Taken together, the contributions included in this Special Issue confirm that Brain Stimulation is a vivid and rapidly expanding field. This perspective is consistent with the European College of Neuropsychopharmacology Manifesto, which proposed NIBS as a new super-subspecialty and highlighted its potential to move psychiatry beyond purely symptom-based models toward circuit-informed, personalized, and mechanism-driven interventions [8]. In this framework, the promotion of standardized training in NIBS appears essential, especially for early-career psychiatrists, to ensure appropriate protocol optimization and safety monitoring, as well as the responsible translation of neuromodulation into clinical practice.
